# Supplementary material for: Role and mechanism of NCAPD3 in promoting malignant behaviors in gastric cancer
Source: Front Pharmacol. 2024 Apr 22;15:1341039. doi: 10.3389/fphar.2024.1341039 (PMC11070777; doi:10.3389/fphar.2024.1341039)
Supplement: Supplementary file 11 [file DataSheet2.ZIP › GSEA/Canonical pathways/my_analysis.Gsea.1599462267220/REACTOME_TRANSPORT_OF_SMALL_MOLECULES.html]

Details for gene set REACTOME\_TRANSPORT\_OF\_SMALL\_MOLECULES[GSEA]

|  || Dataset | filtered\_dataset.sample\_info.cls#WT\_versus\_NCAPD3\_MUT |
| Phenotype | sample\_info.cls#WT\_versus\_NCAPD3\_MUT |
| Upregulated in class | NCAPD3\_MUT |
| GeneSet | REACTOME\_TRANSPORT\_OF\_SMALL\_MOLECULES |
| Enrichment Score (ES) | -0.33111766 |
| Normalized Enrichment Score (NES) | -2.1932576 |
| Nominal p-value | 0.0 |
| FDR q-value | 0.0116817355 |
| FWER p-Value | 0.031 |
Table: GSEA Results Summary

  

Fig 1: Enrichment plot: REACTOME\_TRANSPORT\_OF\_SMALL\_MOLECULES      
 Profile of the Running ES Score & Positions of GeneSet Members on the Rank Ordered List

  

| SYMBOL | TITLE | RANK IN GENE LIST | RANK METRIC SCORE | RUNNING ES | CORE ENRICHMENT || 1 | 3954 | LETM1 | 47 | 0.924 | -0.0083 | No |
| 2 | 1497 | CTNS | 58 | 0.883 | 0.0097 | No |
| 3 | 55343 | SLC35C1 | 92 | 0.804 | 0.0084 | No |
| 4 | 817 | CAMK2D | 165 | 0.710 | -0.0245 | No |
| 5 | 55240 | STEAP3 | 169 | 0.710 | -0.0064 | No |
| 6 | 5701 | PSMC2 | 244 | 0.636 | -0.0428 | No |
| 7 | 528 | ATP6V1C1 | 254 | 0.625 | -0.0316 | No |
| 8 | 538 | ATP7A | 305 | 0.588 | -0.0516 | No |
| 9 | 7220 | TRPC1 | 328 | 0.576 | -0.0514 | No |
| 10 | 197259 | MLKL | 346 | 0.569 | -0.0476 | No |
| 11 | 55676 | SLC30A6 | 353 | 0.565 | -0.0359 | No |
| 12 | 1080 | CFTR | 366 | 0.557 | -0.0288 | No |
| 13 | 5718 | PSMD12 | 372 | 0.552 | -0.0167 | No |
| 14 | 1183 | CLCN4 | 373 | 0.551 | -0.0008 | No |
| 15 | 23315 | SLC9A8 | 398 | 0.537 | -0.0032 | No |
| 16 | 801 | CALM1 | 424 | 0.518 | -0.0068 | No |
| 17 | 6507 | SLC1A3 | 464 | 0.496 | -0.0214 | No |
| 18 | 5245 | PHB | 481 | 0.486 | -0.0193 | No |
| 19 | 8454 | CUL1 | 492 | 0.477 | -0.0130 | No |
| 20 | 8737 | RIPK1 | 493 | 0.477 | 0.0006 | No |
| 21 | 81031 | SLC2A10 | 539 | 0.453 | -0.0197 | No |
| 22 | 5567 | PRKACB | 595 | 0.426 | -0.0481 | No |
| 23 | 286410 | ATP11C | 637 | 0.405 | -0.0668 | No |
| 24 | 23250 | ATP11A | 664 | 0.395 | -0.0747 | No |
| 25 | 51000 | SLC35B3 | 740 | 0.355 | -0.1199 | No |
| 26 | 57181 | SLC39A10 | 811 | 0.313 | -0.1627 | No |
| 27 | 7037 | TFRC | 824 | 0.303 | -0.1629 | No |
| 28 | 6558 | SLC12A2 | 839 | 0.276 | -0.1653 | No |
| 29 | 360 | AQP3 | 857 | -0.263 | -0.1703 | No |
| 30 | 144195 | SLC2A14 | 872 | -0.283 | -0.1725 | No |
| 31 | 6515 | SLC2A3 | 873 | -0.283 | -0.1644 | No |
| 32 | 6520 | SLC3A2 | 878 | -0.290 | -0.1591 | No |
| 33 | 8140 | SLC7A5 | 890 | -0.305 | -0.1585 | No |
| 34 | 3949 | LDLR | 929 | -0.353 | -0.1764 | No |
| 35 | 9388 | LIPG | 937 | -0.361 | -0.1713 | No |
| 36 | 4864 | NPC1 | 964 | -0.384 | -0.1795 | No |
| 37 | 4645 | MYO5B | 985 | -0.395 | -0.1829 | No |
| 38 | 57552 | NCEH1 | 1020 | -0.419 | -0.1961 | No |
| 39 | 10681 | GNB5 | 1105 | -0.481 | -0.2443 | No |
| 40 | 54407 | SLC38A2 | 1182 | -0.553 | -0.2847 | No |
| 41 | 23657 | SLC7A11 | 1205 | -0.581 | -0.2843 | No |
| 42 | 23461 | ABCA5 | 1206 | -0.583 | -0.2676 | No |
| 43 | 861 | RUNX1 | 1293 | -0.687 | -0.3114 | Yes |
| 44 | 8501 | SLC43A1 | 1296 | -0.690 | -0.2931 | Yes |
| 45 | 5046 | PCSK6 | 1297 | -0.699 | -0.2731 | Yes |
| 46 | 57348 | TTYH1 | 1306 | -0.710 | -0.2587 | Yes |
| 47 | 57835 | SLC4A5 | 1319 | -0.736 | -0.2464 | Yes |
| 48 | 6262 | RYR2 | 1323 | -0.741 | -0.2274 | Yes |
| 49 | 10349 | ABCA10 | 1326 | -0.747 | -0.2074 | Yes |
| 50 | 6509 | SLC1A4 | 1330 | -0.752 | -0.1881 | Yes |
| 51 | 7779 | SLC30A1 | 1332 | -0.755 | -0.1672 | Yes |
| 52 | 6646 | SOAT1 | 1333 | -0.755 | -0.1455 | Yes |
| 53 | 9990 | SLC12A6 | 1379 | -0.922 | -0.1524 | Yes |
| 54 | 6541 | SLC7A1 | 1388 | -0.981 | -0.1301 | Yes |
| 55 | 2040 | STOM | 1389 | -0.988 | -0.1018 | Yes |
| 56 | 493 | ATP2B4 | 1401 | -1.166 | -0.0765 | Yes |
| 57 | 6519 | SLC3A1 | 1404 | -1.195 | -0.0437 | Yes |
| 58 | 760 | CA2 | 1410 | -1.654 | 0.0000 | Yes |
Table: GSEA details [plain text format]

  

Fig 2: REACTOME\_TRANSPORT\_OF\_SMALL\_MOLECULES      
 Blue-Pink O' Gram in the Space of the Analyzed GeneSet

  

Fig 3: REACTOME\_TRANSPORT\_OF\_SMALL\_MOLECULES: Random ES distribution      
 Gene set null distribution of ES for **REACTOME\_TRANSPORT\_OF\_SMALL\_MOLECULES**
